# Sense of coherence, spontaneous brain activity, and burnout severity

**DOI:** 10.1192/j.eurpsy.2021.1953

**Published:** 2021-08-13

**Authors:** S. Tei, J. Fujino

**Affiliations:** 1 Department Of Psychiatry, Kyoto University, Kyoto, Japan; 2 Institute Of Applied Brain Sciences, Waseda University, Saitama, Japan; 3 School Of Human And Social Sciences, Tokyo International University, Saitama, Japan; 4 Department Of Psychiatry And Behavioral Sciences, Tokyo Medical and Dental University, Tokyo, Japan

**Keywords:** sense of coherence, burnout, medical professionals, fMRI

## Abstract

**Introduction:**

Burnout has become a critical issue in health care systems during the COVID-19 pandemic. Several studies report on the importance of peoples’ sense of coherence (SOC) or control over work for dealing with burnout. SOC implicates a stress-coping capacity involving comprehensibility, manageability, and meaningfulness. However, little is known on how SOC cognitively modulates burnout experiences.

**Objectives:**

To investigate neurocognitive mechanisms of SOC and burnout in medical professionals.

**Methods:**

Forty-one registered nurses were enrolled. We used functional magnetic resonance imaging and measured resting-state brain activity. We identified brain regions associated with SOC and burnout levels by correlating these trait scores to regional fractional amplitude of low frequency fluctuations (fALFF). Subsequently, we investigated whether participants’ levels of SOC impacted their fALFF-burnout association by mediation analysis.

**Results:**

SOC and depersonalization dimension of burnout were negatively correlated (p < 0.01). The fALFF in the mid-dorsolateral prefrontal cortex (DLPFC) correlated positively with SOC scores, and negatively with depersonalization dimension of burnout (p < 0.05). Furthermore, SOC mediated the negative relationship between DLPFC activity and burnout severity (p < 0.05).

**Conclusions:**

Our data suggested that SOC alleviates burnout experience and supports prefrontal activity to prompt cognitive control; they may facilitate flexible shifting of perspective and optimistic reappraisal of work-stress. In effect, workplace-stressors may be acknowledged as being more meaningful than distressing. Without sufficient SOC, frequent exposures to stressors can lead to maladaptive coping to exhibit emotional numbing or depersonalization.

**Disclosure:**

No significant relationships.

